# Overcoming barriers and enhancing facilitators to COVID-19 vaccination in the Hispanic community

**DOI:** 10.1186/s12889-022-14825-y

**Published:** 2022-12-20

**Authors:** Ramey Moore, Martha O. Rojo, Rachel S. Purvis, Luis Paganelli Marin, Judith Yáñez, Sharon Reece, Cheryl Wells, Brittany Vaughn, Pearl A. McElfish

**Affiliations:** 1 College of Medicine, University of Arkansas for Medical Sciences Northwest, 1125 N. College Ave, Fayetteville, AR 72703 USA; 2 College of Nursing, University of Arkansas for Medical Sciences, 4301 W. Markham St, Little Rock, AR 72205 USA; 3 Office of Community Health and Research, University of Arkansas for Medical Sciences Northwest, 1125 N. College Ave, Fayetteville, AR 72703 USA; 4 RootED Northwest Arkansas, 610B, E. Emma Ave, Springdale, AR 72764 USA

**Keywords:** Hispanic, Latino, COVID-19, Vaccines, Health disparities

## Abstract

**Background:**

Hispanic communities in the United States have been disproportionately affected by COVID-19 infections, hospitalizations, and death. Vaccination against COVID-19 is critical for controlling the pandemic; however, higher levels of vaccine hesitancy and reduced vaccine uptake constrain efforts to mitigate the pandemic and could perpetuate disparities. The aim of this study was to understand barriers and facilitators to COVID-19 vaccination through the lived experiences of Hispanic persons living in Arkansas.

**Methods:**

Bilingual community partners facilitated recruitment, made initial contact with potential participants, and scheduled interviews and focus groups. Individuals over the age of 18 who identified as Hispanic were invited to participate. Data was collected from 49 participants in 10 individual interviews and five focus groups. This study used a qualitative exploratory design and thematic analysis.

**Results:**

Five themes emerged as barriers for Hispanic participants: *technological literacy and pre-registration, language and literacy, health insurance/health care costs, immigration status,* and *location and transportation*. Three themes emerged as facilitators: *workplace vaccination, health care provider recommendations,* and *engagement through schools.*

**Conclusions:**

Based on the findings of this study, a multi-modal and flexible approach will be implemented by the authors to address barriers to vaccine uptake among the Hispanic community in Arkansas.

**Supplementary Information:**

The online version contains supplementary material available at 10.1186/s12889-022-14825-y.

## Introduction

Hispanic communities in the United States (US) have been disproportionately affected by COVID-19 infections, hospitalizations, and death [1–6]. COVID-19 disparities are due in part to lower health insurance coverage, decreased access to health care, and housing and occupational risk factors compared to non-Hispanic White populations in the US [5, 7, 8]; Hispanic populations have the lowest rates of insurance coverage of all racial/ethnic groups in the US, experience concomitant issues of access to care, frequently work in positions without remote work options or paid leave, and often live in larger multi-generational homes that do not allow for social distancing or safe quarantining [6]. Hispanic communities have also experienced significant economic disparities from the COVID-19 pandemic including higher levels of food insecurity, housing disruption, and job loss [7–9].

Vaccination against COVID-19 is critical for controlling the pandemic [10]; however, higher levels of vaccine hesitancy and reduced vaccine uptake constrain efforts to mitigate the pandemic and could perpetuate COVID-19 disparities. Hispanic community members report being more hesitant about the COVID-19 vaccine than non-Hispanic White populations [11], and COVID-19 vaccine uptake has also been slower for Hispanic persons [12, 13].

According to the Increasing Vaccination Model (IVM), vaccination is influenced by a multi-faceted process, which includes social and cultural context, individual beliefs and reasoning, and practical barriers and facilitators [14, 15]. While several quantitative studies document disparities in COVID-19 vaccine hesitancy and uptake [16–18], Hispanic communities were often not the focus of these studies. Additionally, there is limited qualitative research on understanding the reason for disparities in vaccine hesitancy [19, 20]. The current focus of the qualitative literature often combines research on other minority communities with Hispanic participants [21, 22], focuses exclusively on social structural issues [20], or exclusively examines beliefs related to COVID-19 vaccination [19]. This study fills a gap by focusing exclusively on Hispanic community members to understand barriers and facilitators to COVID-19 vaccination.

Understanding the causes of vaccine inequalities, and specifically the barriers and facilitators of vaccination for Hispanic communities, is critical for developing evidence-based strategies for increasing vaccine uptake and reducing health disparities faced by these communities [6]. The aim of this study was to understand barriers and facilitators to COVID-19 vaccination through the lived experiences of Hispanic persons living in Arkansas.

## Methods

### Study approach and design

This study used a qualitative exploratory design and thematic analysis [23, 24] to understand barriers and facilitators to COVID-19 vaccination among the Hispanic communities living in Arkansas. All study materials and procedures were approved by the University of Arkansas for Medical Sciences Institutional Review Board (IRB #261965).

A community-based participatory research (CBPR) approach was used in the design and implementation of the study. CBPR honors cultural values, social practices, and community norms and integrates communities into every aspect of the research [25, 26]. Community partners provided input on the study aims and design, facilitated participant recruitment, assisted with data collection, and provided feedback on findings during data analysis. The CBPR collaborative is described in several articles [7].

### Participant recruitment, consent, and remuneration

Potential participants were recruited between February 2021 and June 2021. Bilingual community partners facilitated recruitment, made initial contact with potential participants, and scheduled interviews and focus groups. Individuals over the age of 18 who identified as Hispanic were invited to participate. Participants’ consent was obtained in their language of preference (English or Spanish). Research Electronic Data Capture (REDCap) was used to record participant consent [27]. Participants received a $40 gift-card for participation in the study.

### Data collection

Individual interviews and focus groups were conducted between February 2021 and July 2021 via a secured video conferencing platform and telephone [28]. Most interviews and focus groups were conducted in Spanish; one focus group was conducted in English at the request of participants. All interviews were conducted by bilingual members of the research team trained in qualitative interviewing. During the focus group, an additional research team member was present to take notes.

Data was collected from 49 participants in 10 individual interviews and five focus groups. The focus groups consisted of two to 12 participants and lasted 60 to 120 minutes. The individual interviews ranged in duration from 20 to 60 minutes. The individual interviews and focus groups were recorded, professionally transcribed, and de-identified, and Spanish transcripts were translated from Spanish to English by certified Spanish translators. Bilingual study staff verified transcripts using audio recordings prior to analysis.

### Qualitative data analysis

Analysis was conducted using MAXQDA 2020. Three qualitative researchers read and analyzed all responses and created a codebook with emergent codes. Segments of text were coded by the first author, with confirmation-coding analysis performed by two additional qualitative researchers. Initial codes were refined, and the codebook was revised four times. Codebook iterations were used to reach consensus on codebook structure, emergent themes, and thematic domains and to verify data saturation. Any differences in interpretation of data were discussed by the research team and resolved using a consensus model. Illustrative quotes were identified to illustrate thematic domains. Participants often indicated several motivating factors within a single answer, and all quotes are presented within the themes they best represent. The research team critically reviewed the data, analysis summaries, and codebook. They then coded segments to ensure analytic rigor, reliability, and full data saturation [29]. Descriptive sociodemographic statistics were summarized for the study sample to show frequencies and percentages.

### Instrument

A semi-structured interview guide was developed collaboratively with community partners to ensure the questions were culturally relevant, informed by the community, and written in plain language. The interview guide developed for this study is provided as Additional file 1. The interview guide was revised and translated into Spanish and then reviewed by two additional Spanish language translators. The interview guide included questions on barriers and facilitators of vaccination decisions. In addition to the qualitative interview guide, sociodemographic factors were captured using questions from the Behavioral Risk Factor Surveillance System (BRFSS) survey [30]. Participants were asked to report their age, race and ethnicity, level of education, and employment type and income.

## Results

Sociodemographic characteristics of the sample are presented in Table 1. All participants (*n* = 39) identified their ethnicity as Hispanic. A majority of participants were female (69.2%). Most participants reported some high school or attainment of a high school diploma (64.8%). Most participants reported they were unvaccinated at the time of their participation (87.2%).

**Table 1:** Demographics

Total Participants (*n* = 39)	
Category	
Sex	
Male	12 (30.8%)^a^
Female	27 (69.2%)
Education	
< HS	14 (37.8%)
HS	10 (27.0%)
Some college	4 (10.8%)
College +	9 (24.3%)
Vaccinated	
Yes	5 (12.8%)
No	34 (87.2%)

*HS* High school

^a^Percentages may not add up to 100% due to rounding

Hispanic participants reported a range of issues that served as barriers or facilitators to vaccination against COVID-19. Qualitative themes are presented in Table 2. Five themes emerged as barriers: *Technological literacy and pre-registration, language and literacy, health insurance/health care costs, immigration status,* and *location and transportation*.

**Table 2:** Themes

Primary Themes	Secondary Themes	Sub-Themes
Barriers to vaccination	Technological literacy and pre-registration	• Online appointment booking technologies
		• Pre-registration needed for vaccination
	Language and literacy	• Institutionalization of English
		• Lack of translation into Spanish
		• Literacy
		• Plain language translation
	Health insurance/health care costs	• Lack of health insurance
		• Uncertainty concerning cost of care
		• Synergistic barrier alongside immigration status
	Immigration status	• Lack of documentation
		• Appointments/providers require SSNs or other
		• Fears that immigration status will be reported
	Location and transportation	• Information about vaccination locations
		• Convenience
		• Transportation
Facilitators to vaccination	Workplace vaccination	• Hosting on-site vaccination clinics
	Health care provider recommendations	• Vaccination messaging from local clinics and bilingual physicians
		• One-on-one conversations with health care providers
	Engagement through schools	• School system as trusted source of information

*SSN* Social security number

### Technological literacy and pre-registration

The pre-registration process and requirements to use technology to pre-resister were described as primary barriers, which were interrelated with language and immigration barriers. Participants frequently identified barriers related to the requirements for scheduling vaccination appointment via phone or internet technologies. Pre-registration systems were the focus of participant discussion of barriers to vaccination. Participants explained, “When it comes to the appointment, I’m not that good with the phone or using the internet, so it’s very hard for me to make an appointment. Because they say that they only do it online.” Another participant related her sister’s experience with technology as a barrier to vaccination. She stated, “They want you to make an appointment over the phone and many, many people don’t know how to make the appointment over the phone. For example, my sister does not use the phone. She has to go in person.” Other participants noted that many in the Hispanic community “don’t know how to use the internet” and how this lack of technological literacy is related to low levels of vaccination in the community.

Participants described how even after deciding to get vaccinated, they were turned away because they did not pre-register. Participants recommended: “I think that it would be nice if there were a center for—where there is—where there is attention for—face to face, I mean. Where there’s someone who can take care of you, and, well, if anything, to say [ …] if possible, give them the vaccine right there.”

### Language and literacy

The institutionalization of English as the primary language for delivering information about vaccines was also described as a barrier by Hispanic participants. Participants stated: “I tried the other day, and I didn’t - didn’t find anything of that in Spanish, so all that becomes very complicated,” and another participant stated, “Many times [ …] when people arrive to the place where they are vaccinating, there are only people who speak English and if I don’t speak English, how can I go and ask [ …] how am I going to get the vaccine?” Participants continued to identify language as a primary barrier: “Living in a country where the language that is spoken is not our own. We are finding ourselves with a barrier where we don’t speak, or we don’t know how to read it,” and another stated, “My family, they do speak Spanish and they write it, but not English. We struggle in this country because, well many times you can’t buy or acquire what you need because of English because you do not know it.” Participants described the struggles with overcoming language barriers: “One tries to defend yourself, by trying to speak a little in English. There you take out the translator on your phone, or something, and well, at the end of the day you get it done but it is difficult.”

Participants went on to discuss how literacy, in addition to language, was a barrier. One participant described how illiteracy increased the difficulty of tasks, such as vaccination: “It’s hard, uh, yes, I can speak and can write in Spanish, and I think you can do lots of things but imagine how the people who don’t know how to read or write. It must be harder.” Another participant described the way language and literacy influence access:

I think sometimes, that is the problem. [ …] When we speak technically or on an elevated level academically, we lose interest. We just simply say, “I didn’t end up learning, I don’t know anything.” But, to add onto that, we should find places, in this case like [a local non-profit community organization], to try to disseminate the information in our language, but also on our level.

Other participants echoed that translation into Spanish was not enough and explained that information about the vaccine at vaccination sites, or in clinics, must be translated into comprehensible, everyday language. Participants stated, “I would like to simply—I think, I think we’d all like to receive information in Spanish, in our—in all our levels of Spanish, but have it be a Spanish that … how we all speak so that we can all understand. Not these big words.”

### Health insurance/health care costs

While COVID-19 vaccination is free, participants described uncertainty about the cost of COVID-19 vaccines and lack of health insurance as barriers to vaccination for Hispanic participants. Participants described this situation in detail, stating:

It could also be that they do not have insurance or money to apply the vaccine, for example [in local city] they had a stand for vaccination, and it looked like it was free but upon arrival if you did not have any insurance, they were charging up to a hundred dollars and from there, there isn’t much you can do if you don’t have the hundred dollars, so I think that this is also a big barrier. If someone doesn’t have insurance or access to a doctor, this is the barrier that will have people believing they can’t go out or that they won’t have access to it.

Several participants identified insecurity about potential costs as barriers to COVID-19 vaccination, especially for those who are uninsured. One participant described how the lack of medical insurance “generates a big barrier,” which was particularly an issue for her and others in the community, “because those of us who live day to day, what I earn today I spend today because I use it for groceries, to save up for rent, and things like that.” Participants described how even the fear of potential costs was a significant barrier: “[It] makes me feel that it’s impossible for me to sign up to, or to stand in line so that they can give me the, the vaccine.” Another participant described how the lack of insurance causes fear concerning medical care: “There are people that are afraid of hospitals and clinics, and there are people, because they lack insurance, they can’t [seek vaccination] because of fear.”

Acting synergistically, participants often discussed the lack of health insurance alongside issues related to immigration status. One participant described how there “are a lot of people that for the fact that they don’t have insurance or are not with the social security, well, they are not going to get it. And-and then, well, if they are being asked insurance, well, anyway, they don’t have an option.” Another participant narrated the experiences of a member of her extended family:

[My family member] didn’t know if [staff at a vaccination site] were asking if he has social security or insurance. And he told me, ‘No, they only ask me for my number—that, that they need my security number, and I hung up.’ He said, ‘I don’t have anything, what was I supposed to give them?’ He doesn’t have either social security or private insurance. And that is something, eh, we, Hispanic people and those without insurance are afraid.

### Immigration status

Immigration status was frequently cited as a barrier to vaccination for members of the Hispanic community. One participant described immigration status as the “number one” barrier “for those that are illegal and not legal and being afraid of giving out their information and saying they’re going to come get me because I just gave them my information when I got the vaccine.” Another participant described how the lack of legal status and documentation affects vaccination uptake in the Hispanic community: “I think that people who don’t have their card, or are not legal residents, those people I think are afraid and they might have trouble**.** There are those that are afraid because of their legal status, [ …] they think that this is another way to send them back to their countries.”

Participants described how the registration process often requires a social security number (SSN). As one participant narrated their experience, “I tried to sign up my parents for [an appointment for the COVID-19 vaccine] and like, in the forms, if you try to sign up online in the form [ …] they are asking for your social and I’m like *if* you have a social.” Even for vaccination sites that do not specifically require an SSN, undocumented Hispanic residents express fear about the information entered into computer systems. One participant described this situation: “A lot of people, we are afraid to write down all our information [ …] in the website, [or] in a mobile device, or in a computer because you don’t know who is on the other side of the computer, or who’s monitoring all that, or who’s looking at all that [and] you don’t know, uh, who has access to the information.”

### Location and transportation

Participants described a lack of information about the vaccine locations, a lack of convenient locations, and a lack of transportation to get to vaccination locations as significant barriers. Participants described a lack of information about exact vaccination locations and how that caused uncertainty: “‘You’re coming to Walmart.’ But where? […] where do I have to go? To the pharmacy? To next to Walmart, or, well, over to the office? I don’t know where you could go to get the vaccine.” Participants described the need for quick and convenient locations for vaccination.

In this country [ …] you have a job, you have two jobs, and many people have up to three jobs, and [ …] there’s not enough time to say, like, ‘Oh, I have all day today to - to go get the vaccine.’ I think that at a center—a place where, practically, it’s on your way, you say, ‘let me get the vaccine. Uh, I’ll stop by right now.’

Transportation to and from vaccination sites was identified a barrier when seeking to get to current vaccination sites for Hispanics. Participants described a range of transportation related issues, including how immigration status overlaps with other transportation barriers.

Another thing is like getting there, you know. Some of these families [ …] didn’t have a way of getting around. Even if we needed the resources, and even if we would have known, we probably wouldn’t have been able to get there. And we do live in an area where it’s not like you can just hop on the bus and go. [ …] And some of these Hispanics in the Hispanic community, some of them can’t drive ‘cause you know, you know they’re not legal.

Other participants described how difficult it is to “travel far to get [vaccinated],” especially for “the people that are old or don’t have a way to travel like transportation.” Another Hispanic woman noted “some women do not drive very much,” which is compounded by a lack of transportation options for those who don’t drive or have easy access to a vehicle. She continued comparing living in Arkansas to the experiences of a friend in an urban area: “My friend [ …] can use a cab, right, if you want to go anywhere [ …] but let’s say she lived here, where you need to have a car to go anywhere?”

Three themes emerged as facilitators: *workplace vaccination, health care provider recommendations,* and *engagement through schools.*

### Workplace vaccination

Employer support for vaccination was an important facilitator for many participants, especially when employers hosted vaccination events on-site during work hours. One participant described how receiving a vaccination at work addressed two barriers: the pre-registration for appointment and language barriers in filling out required paperwork. This participant stated, “I think that at work is the best, because there you don’t need an appointment, [and] there they help you fill out paperwork, you fill out a form and they tell you when you will receive it, and sometimes it is the next day.” Participants connected on-site vaccination with both convenience and caring for their employees: “They are providing the vaccine there at the plant, so I think I would like to have the company I work for do the same [ …] I would like to know that they care for their workers, and they can help us at work too.” Some participants noted workplace vaccination opportunities, even when not mandatory, made it possible for them to be vaccinated: “It wasn’t mandatory at work, but the opportunity was there, and they offered both doses. I did take both.” Another participant echoed this sentiment and noted many co-workers who were hesitant did make the decision to be vaccinated at work, describing how his hesitant co-workers “made up their minds [ …] they wanted to get vaccinated. But, I think that the sensitive thing was–that the company allowed [ …] for them to make the decision of getting vaccinated.”

### Health care provider recommendations

Health care provider’s recommendation was identified as an important facilitator for vaccination. Participants noted clinics and doctors’ offices were respected in the Hispanic community, and advice from a trusted physician could result in vaccine-seeking behavior. One participant described how her mother’s physician influenced her decision to be vaccinated: “I honestly believe this is going to be safe for my mom, sister and myself mainly because her doctor has told her not to waste any opportunity in getting the vaccine.” Another participant expanded on this: “Her mom’s doctor had given her good advice about the vaccine and that is what we all need [ …] information from someone who works in health care and can speak well about the vaccine.” This participant concluded, “I would feel so much better if a doctor or a professional told me this. Do I need the vaccine? Should I wait? I feel this would push me to get vaccinated if I hear it from someone like that.”

Participants described the value of conversations with health care providers: “I think one of the key factors [would be] having their doctors explain [COVID-19 vaccines] to them [and] having a professional that you know and like. I think that would be helpful having the doctors [say], ‘Hey let me talk to you a little bit more about the vaccine now that you’re here for your yearly checkup.’” Participants also recommended health care providers ask about vaccination status and then address their patients’ specific concerns: “[The doctor should] see if you’ve already had it or [ask] ‘what can I do … what’s the questions that you had?’ Just having that one-on-one [ …] answering questions with them would be helpful.” Another participant expanded on this idea, stating that they would be more likely to receive the COVID-19 vaccine with more information from a health care provider “if the nurse or the doctor who will be giving you the vaccine goes and says, ‘look [identifier] this is what you’re going to receive, this is what you will, the side effects, [this is] what they’re actually putting in your body.’ Because I don’t know the contents of the vaccine.”

### Engagement through schools

Engagement through schools as trusted social institutions was described as a potential facilitator for increasing vaccine uptake within Hispanic communities. As one participant suggested, “Think of using the school, parents have to go to the school and, well, I was thinking maybe the fathers and mothers can get information while waiting for their children. Places that people trust.” Another participant suggested using schools and how this information would be understood by members of the Hispanic community:

Use the schools, right? Through schools. It’s the main instrument. Many times, they, the children, they sometimes can reflect on what they learn, they manage to understand—to reflect more than an adult can do, right? [ …] Sometimes children bring the information to our homes and it is more acceptable. Schools can serve as bridges to reach our home. Schools can be used to deliver information to households.

Another participant noted that the schools provide an avenue to reach households who lack health care access: “I mean, for me the best information is in the clinic, but there are a lot of people who don’t go to the clinic. They only go when it’s necessary.” The participant recommended providing information “at the children’s schools [which] is a good medium to get it at least to the parents and from there, you know, the chain starts to form and it starts, ‘look, I got this from the child’s school about a vaccine,’ I mean, that’s, that’s how it starts.”

## Discussion

This study explored barriers and facilitators to COVID-19 vaccination among the Hispanic community in Arkansas. Five barriers to vaccination were identified: technological literacy and pre-registration, language and literacy, health insurance/health care costs, immigration status, and location and transportation. Participants described three facilitators that made it easier to become vaccinated against COVID-19: workplace vaccination, health care provider recommendations, and engagement through schools.

The requirement to pre-register and the use of technology used to pre-register were described as a barrier to vaccination. This is the first study to document the pre-registration process itself as a barrier for the Hispanic community; however, our findings are consistent with the literature showing adverse effects of the ‘digital divide’ and lower levels of computer and internet literacy on vaccine-seeking behaviors in Hispanic communities [31].

Language and literacy were critical barriers for Hispanic participants. This finding is consistent with prior research that shows language barriers are commonly identified as preventing access to a variety of health services [32, 33], including the COVID-19 vaccine [34, 35]. The present study adds important new information showing Spanish translation is not sufficient to meet the needs of the community and that it is critical to provide information in plain language for low literacy levels [36].

A key finding of this study is that lack of health insurance and fear of costs are barriers to COVID-19 vaccine uptake in Hispanic communities. Hispanic community members have the lowest rates of health insurance coverage among all racial and ethnic groups in the US [6]. For participants in this study, lack of health insurance and the fear of cost were identified as barriers even though the COVID-19 vaccine is provided at no cost. This finding builds on our emerging understanding that fear of out-of-pocket costs is a barrier for the uninsured, especially for non-citizen immigrants [37].

Immigration status and fear of deportation were reported as major barriers to COVID-19 vaccination uptake among Hispanic communities. Immigration policies have direct and indirect effects on Hispanic health care utilization and health outcomes [38, 39], with insecurities around immigration status shown to shape vaccine-seeking behaviors [20]. Our findings are consistent with emerging literature on the COVID-19 vaccine in Hispanic communities in the US linking undocumented status and fears of immigration status disclosure to lowered rates of vaccination uptake [37, 40].

Vaccination location, lack of clear information about the location, and transportation were described as barriers to vaccination for the Hispanic community. This finding is consistent with prior literature which highlights how the lack of clear information about local vaccination sites compounds disparities in vaccination uptake in racial and ethnic minority communities [37]. Convenient locations for vaccination have been found to be an important determining factor for other vaccines and in at-risk populations, including Hispanic communities [37]. Transportation has been cited as a barrier for health care access generally [41] and for Hispanic communities specifically during the COVID-19 pandemic [34, 42, 43]. As far as the authors are aware, the current study is the first study to explore the synergistic interactions among these particular barriers for Hispanic communities. This study supports the contention that increasing vaccine uptake and addressing hesitancy may require multi-component interventions [44, 45].

Workplaces providing COVID-19 vaccination was identified as a major facilitator. Participants described how employers who provided on-site vaccination clinics made it easier to make the decision about vaccination and made it easier to receive the COVID-19 vaccine. Our finding that workplace support for vaccination increases vaccine uptake is novel and points to the need for further implementation and investigation of workplace interventions to increase COVID-19 vaccination.

Participants identified recommendations from primary care health care providers who answered questions about the COVID-19 vaccine as a critical facilitator. This finding is consistent with emerging research on the importance of health care provider communication in facilitating COVID-19 vaccine uptake [46–49]. Unfortunately, Hispanic community members are less likely to report having a primary care provider [50]. Participants also identified the school system as a facilitator for vaccination through distributing reliable information about the COVID-19 vaccines. This is consistent with the literature on the role of educational institutions in distributing public health messaging to increase vaccine uptake [19, 51].

Our findings highlight the importance of the structural, logistical, social, political, and cultural contexts of receiving the COVID-19 vaccine among Hispanic community members. Long-term and ongoing factors affecting Hispanic community health had significant effects on inequalities in health outcomes during the COVID-19 pandemic and, importantly, also affected efforts to mitigate these effects through vaccine uptake in Hispanic communities. This study demonstrates the need to address these social determinants of health for the long-term benefit of Hispanic communities and overall public health in the US, as well as targeted application of community-based and culturally sensitive interventions to improve COVID-19 vaccine uptake in these communities.

### Recommendations for practice

Based on the results, the authors and CBPR partners make several recommendations to increase COVID-19 vaccine uptake in Hispanic communities. Recommendations for practice are presented in Table 3. To address barriers related to technological literacy, public health leaders should provide access to vaccinations for walk-up/drive-up that do not require pre-registration. For vaccination sites that require pre-registration, it is imperative the registration processes be simple. Bilingual community health workers (CHW) should be used to facilitate pre-registration and reduce inequalities related to the digital divide.

**Table 3:** Recommendations for policy and practice

**Barriers**	**Recommendations for Practice**
Technological literacy and pre-registration	• Allow for walk-up/drive-up vaccination that does not require pre-registration
	• Ensure pre-registration process is easy
	• Allow for multiple methods for pre-registration
	• Utilize CHWs to help community members with pre-registration
Language and literacy	• Provide information in Spanish at every stage of vaccination process (advertising, COVID-19 vaccine FAQs, and at vaccination sites)
	• Ensure all information is provided for low levels of literacy
	• Utilize Spanish-speaking staff at vaccination sites
	• Utilize CHWs to help community members overcome language and literacy barriers
	• Utilize video communications to overcome literacy barriers
	• Create low literacy templates for Spanish language communications that can be branded by community organizations
Health insurance/health care costs	• Clearly communicate COVID-19 vaccines are available without cost to patients
	• Increase access to health insurance and health care services such as federally qualified health centers who provide care on a sliding fee scale for the uninsured
Immigration status	• Clearly communicate that immigration status will be kept confidential
	• Do not require SSNs or other residency documents in the appointment process or at vaccination sites
Location and transportation	• Offer mobile vaccine events and long-term vaccine location in neighborhoods where Hispanic community members live
	• Provide clear information about the locations in Spanish
	• Offer transportation assistance to get to COVID-19 vaccine locations
	• Utilize CHWs to help find transportation and navigate community members to vaccine locations
**Facilitators**	**Recommendations for Practice**
Workplace vaccination	• Encourage workplace vaccination locations
	• Provide help finding a location
	• Provide paid time off to receive a vaccination
Health care provider recommendations	• Utilize Spanish-speaking health care providers in vaccine outreach campaigns
	• Utilize CHWs to provide information about the COVID-19 vaccine
	• Increase the proportion of the Hispanic community who have a primary care physician
	• Increase diverse health care workforce that includes Hispanic and/or Spanish-speaking health care providers
Engagement through schools	• Implement vaccine information campaigns through schools
	• Provide vaccines through school-based clinics

*CHW* Community health worker, *SSN* Social security number

To address language and literacy barriers, public health leaders should ensure information is provided in Spanish at all stages of the vaccination process, including advertising, COVID-19 vaccine health communication and outreach messaging, and at vaccination sites. In addition, it is critical all information be provided in plain language at low literacy levels, and Spanish-speaking staff should be available at vaccination sites. Bilingual CHWs could also help community members overcome language and literacy barriers. Integrating video into public health messaging can also be implemented to overcome literacy barriers, especially for use on social media platforms. Low literacy communication templates should be created and distributed to community organizations and can be branded with organizational branding to take advantage of these organizations’ position within the Hispanic community.

To address health insurance and health care cost barriers, public health leaders must clearly communicate COVID-19 vaccines are available without cost to patients. Long-term, it is critical to increase access to health insurance and health care services such as federally qualified health centers who provide care on a sliding fee scale for the uninsured.

To address immigration status concerns, public health leaders should clearly communicate immigration status will be kept confidential, and patients should not be required to provide proof of social security number or legal residency in the appointment process or at time of vaccination.

To address location and transportation barriers, public health leaders should offer mobile vaccine events and long-term vaccine location in neighborhoods where Hispanic community members live and provide clear information about the vaccine locations in Spanish. In addition, transportation assistance to COVID-19 vaccine locations is warranted. CHWs may also facilitate transportation assistance to navigate community members to vaccine locations.

To leverage workplaces as a vaccine facilitator, public health leaders should partner with worksites and encourage employer vaccination locations. To leverage the facilitator of health care provider recommendations, public health leaders should utilize Spanish-speaking health care providers in vaccine outreach campaigns and utilize CHWs to provide information about the COVID-19 vaccine. Given the importance of primary care providers in vaccine uptake, it is critical to implement programs that will increase the proportion of the Hispanic community who have a primary care physician. Long-term public health efforts would benefit from a more diverse health care workforce that includes more Hispanic and/or Spanish-speaking health care providers. To leverage the facilitator of engagement through schools, public health leaders should implement vaccine information campaigns through schools and provide vaccines to both children and parents through school-based clinics.

### Strengths and limitations

There are several strengths of the study. First, semi-structured qualitative focus groups and interviews allow key concepts to be explored in depth and in participants’ own words. Additionally, the role of Hispanic facilitators for focus groups and interviews, coupled with allowing participants to conduct data collection in their language of preference (English or Spanish), may increase participants’ candor. This study is not without limitations. This study sample is exclusively from Arkansas and self-selected for participation; thus, findings may not be generalizable across all Hispanic communities in the US, especially among second and third generation Hispanics. Additionally, the larger number of women participants may limit the generalizability of these findings.

## Conclusion

Hispanic communities in the US have been disproportionately affected by COVID-19. Increasing vaccination uptake in Hispanic communities is important for mitigating the effects of COVID-19 disparities. While there were many barriers to COVID-19 vaccine uptake identified by participants, there are ways to leverage facilitators and implement efforts to address the barriers. Based on the findings of this study, a multi-modal and flexible approach will be implemented by the authors to address barriers to vaccine uptake among the Hispanic community in Arkansas.

## Supplementary Information

**Additional file 1: **COVID-19 Vaccine Interview Guide. A semi-structured interview guide was developed collaboratively with community partners to ensure the questions were culturally relevant, informed by the community, and written in plain language. The interview guide was revised and translated into Spanish and then reviewed by two additional Spanish language translators. The interview guide included questions on barriers and facilitators of vaccination decisions.

## Data Availability

The deidentified data underlying the results presented in this study may be made available upon request from the corresponding author, Dr. Pearl A. McElfish, at pamcelfish@uams.edu. The data are not publicly available in accordance with funding requirements and participant privacy.

## Abbreviations

US: United States
IVM: Increasing Vaccination Model
IRB: Institutional Review Board
CBPR: Community-based participatory research
REDCap: Research Electronic Data Capture
BRFSS: Behavioral Risk Factor Surveillance System
SSN: Social security number
CHW: Community health workers
