# Supplementary material for: Overcoming barriers and enhancing facilitators to COVID-19 vaccination in the Hispanic community
Source: BMC Public Health. 2022 Dec 20;22:2393. doi: 10.1186/s12889-022-14825-y (PMC9765355; doi:10.1186/s12889-022-14825-y)
Supplement: Supplementary file 1 — Additional file 1: COVID-19 Vaccine Interview Guide. A semi-structured interview guide was developed collaboratively with community partners to ensure the questions were culturally relevant, informed by the community, and written in plain language. The interview guide was revised and translated into Spanish and then reviewed by two additional Spanish language translators. The interview guide included questions on barriers and facilitators of vaccination decisions. [file 12889_2022_14825_MOESM1_ESM.docx]

**Community Based COVID-19 Testing Audio Recorded Qualitative Questions**

| **COVID-19 Vaccine Interview Guide** |
| --- |

My name is __________________ thank you for participating in this focus group. I (we) want to ask you a few questions regarding the Covid-19 vaccines.

**Grand Tour Question:**

- What have you heard about the COVID-19 vaccines? (specific main tour question)

**Main Questions:**

- Since there are vaccines that could prevent COVID-19, how would you feel about receiving a vaccine yourself?

***Probe: What plans do you have to get a vaccine?***

- What concerns do you have about receiving a COVID-19 vaccine?
- What would make it easier for you to receive a COVID-19 vaccine?

**Probe: *What information would you need to make a decision on taking the vaccine*?**

- What might prevent you from getting a vaccine?
- How would you feel about your family receiving the COVID-19 vaccine?

***Probe: What plans do they have to get a vaccine?***

- What are your concerns about your family receiving a COVID-19 vaccine?

***Probe: Who makes healthcare decisions in your household?***

- What would make it easier for them to receive a COVID-19 vaccine?
- What might prevent your family from getting a vaccine?
- What do you think your community is saying or knows about the vaccine?
- Are there specific actions that health care workers could do to help people decide to receive the vaccine?
- If you were to receive a COVID-19 vaccine, what place would you prefer to receive the vaccine?

*Probe: a) in a large hospital*

*b) in your local community clinic*

*c) In your neighborhood*

*d) Your local pharmacy*

*e) in your place of worship (church)*

*f) in your site of employment*

*g) a health worker coming to your home*

*h) Other*

***Probe: Why is this your preference?***

**Closing question**

Is there anything about the COVID-19 vaccination that I did not ask, such as where you would go to get vaccinated, about your decisions or concerns related to the vaccine, or anything else?
